# Acromegaly in a patient with a pulmonary neuroendocrine tumor: case report and review of current literature

**DOI:** 10.1186/s13104-016-2132-1

**Published:** 2016-06-27

**Authors:** Sebastian Krug, Michael Boch, Peter Rexin, Andreas Pfestroff, Thomas Gress, Patrick Michl, Anja Rinke

**Affiliations:** Department of Gastroenterology, Endocrinology and Metabolism, Philipps-University Marburg, Marburg, Germany; Institute of Pathology, Philipps-University Marburg, Marburg, Germany; Department of Gastroenterology and Hepatology, Martin-Luther University Halle/Wittenberg, Ernst-Grube Straße 40, 06120 Halle (Saale), Germany; Department of Nuclear Medicine, University Hospital Marburg, Marburg, Germany

**Keywords:** Paraneoplastic syndromes, Acromegaly, Pulmonary neuroendocrine tumor, GH, IGF-1

## Abstract

**Background:**

Pulmonary neuroendocrine tumors (NET) form a heterogeneous group of rare diseases. In these tumors, paraneoplastic syndromes have been described to drive the course of the disease, among them acromegaly induced by paraneoplastic secretion of growth hormone-releasing hormone (GHRH).

**Case presentation:**

We report the case of a 43 years old patient initially diagnosed with acromegaly accompanied by weight gain and acral enlargement. Subsequently, further diagnostic work-up identified a solitary pulmonary neuroendocrine tumor (NET). Laboratory tests revealed markedly increased growth hormone (GH) and insulin-like growth factor 1 (IGF-1) without GHRH elevation in the absence of pituitary pathologies confirming the paraneoplastic origin of clinical presentation with acromegaly. Curative surgery was performed leading to normalization of the elevated hormone levels and improvement of the clinical symptoms. Immunohistochemically, a typical carcinoid (TC) was seen with low proliferation index and abundant IGF-1 expression.

**Conclusions:**

The association of acromegaly and pulmonary NET has only rarely been reported. We present an individual case of paraneoplastic GH- and IGF-1 secretion in a patient with pulmonary NET. Based on their rarity, the knowledge of paraneoplastic syndromes occurring in patients with pulmonary NET such as acromegaly due to paraneoplastic GH- and IGF-1 secretion is mandatory to adequately diagnose and treat these patients.

## Background

Pulmonary neuroendocrine tumors (NET) represent rare malignancies with an incidence between 0.2 and 2.0/100.000 inhabitants [1]. The incidence is increasing over the last decades which probably reflects increased awareness of this entity. The literature describes up to 25 % of all neuroendocrine neoplasias (NEN) and approximately 2 % of all lung tumors as pulmonary NET [2–4]. Only few pulmonary NET (5 %) are associated with multiple endocrine neoplasia type 1 (MEN-1) syndrome [5]. Pulmonary NET can be classified into typical carcinoids (TC) and atypical carcinoids (AC) based on clinico-pathological features [4]. TC are 5–10 times more frequently compared to AC. Up to 15 % of TC have already metastasized at time of presentation [6]. Histopathological assessment comprises mitotic-index and necrosis to differ between both subtypes [7]. Moreover, pulmonary NET have been linked to hormone syndromes in up to 30 % of the cases which include carcinoid-, cushing- and inappropriate antidiuretic hormone secretion syndrome as well as rarely acromegaly [4, 8]. Less than 1 % of all acromegaly cases are caused by non-pituitary tumors. In these cases, usually growth hormone (GH) is hypersecreted [9]. This report presents a patient with acromegaly due to rare paraneoplastic GH and IGF-1 secretion mediated by a pulmonary NET.

## Case report

**Fig. 1:**
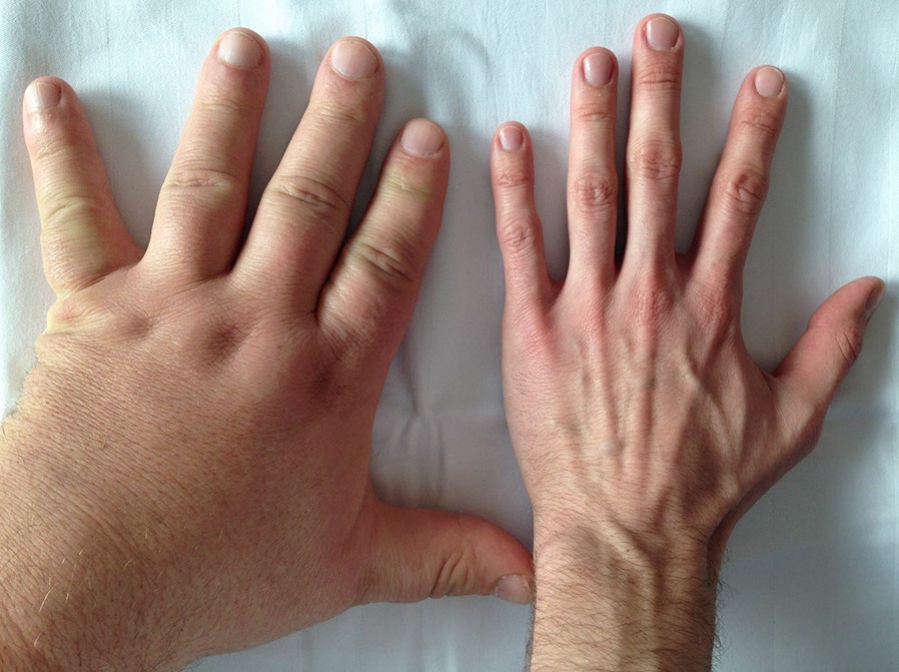
Representative image of the patient’s hand (*left side*) before surgery compared to a hand of one of our doctors

**Fig. 2:**
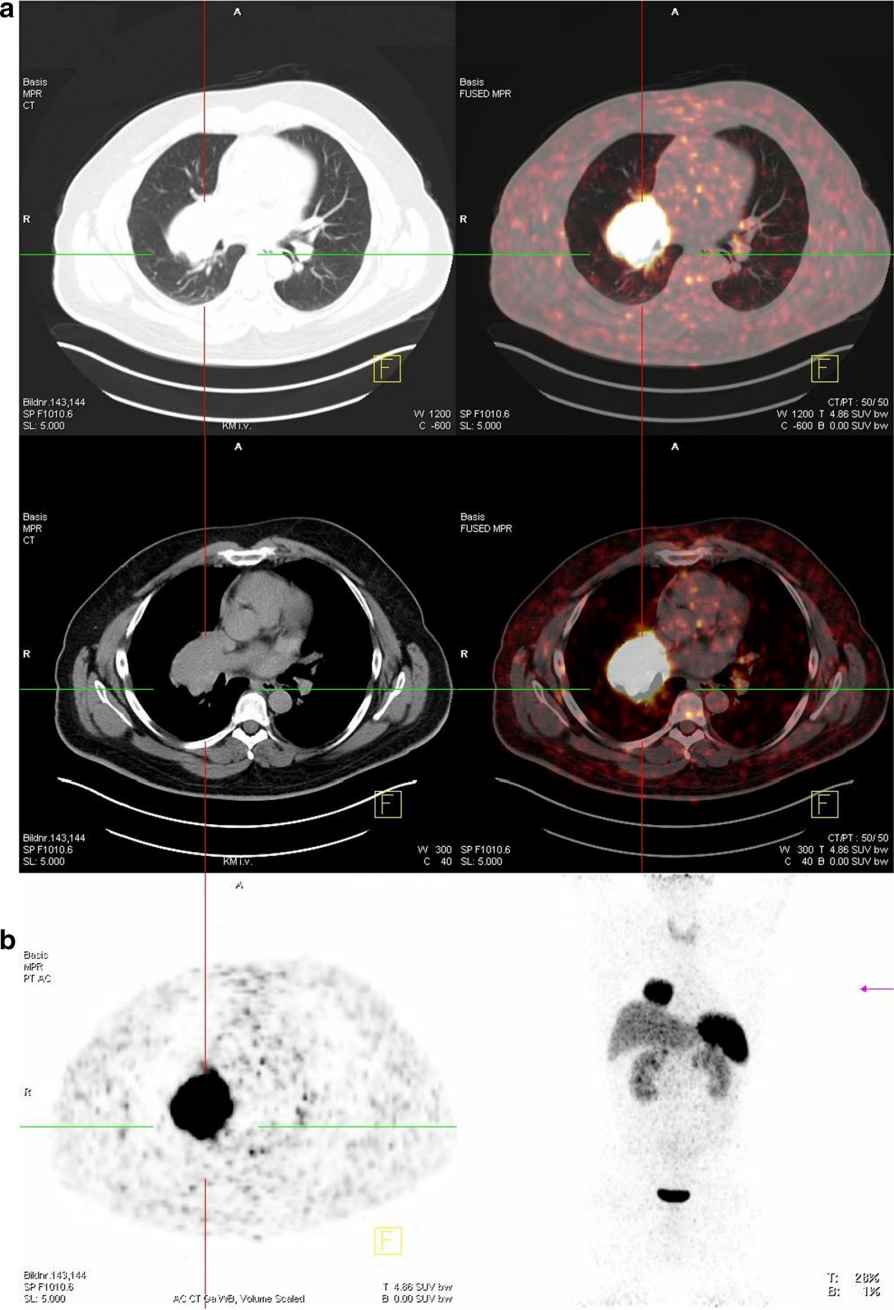
^68^Gallium-DOTATOC PET-CT before surgery revealed a large tumor mass located in the *right* lung with high somatostatin receptor expression. Isolated CT- and PET component (**a** *left side* and **b**) is presented as well as combined images (**a** *right side*). Metastatic spread was excluded as seen on the planar reconstruction (**b** *right side*)

**Fig. 3:**
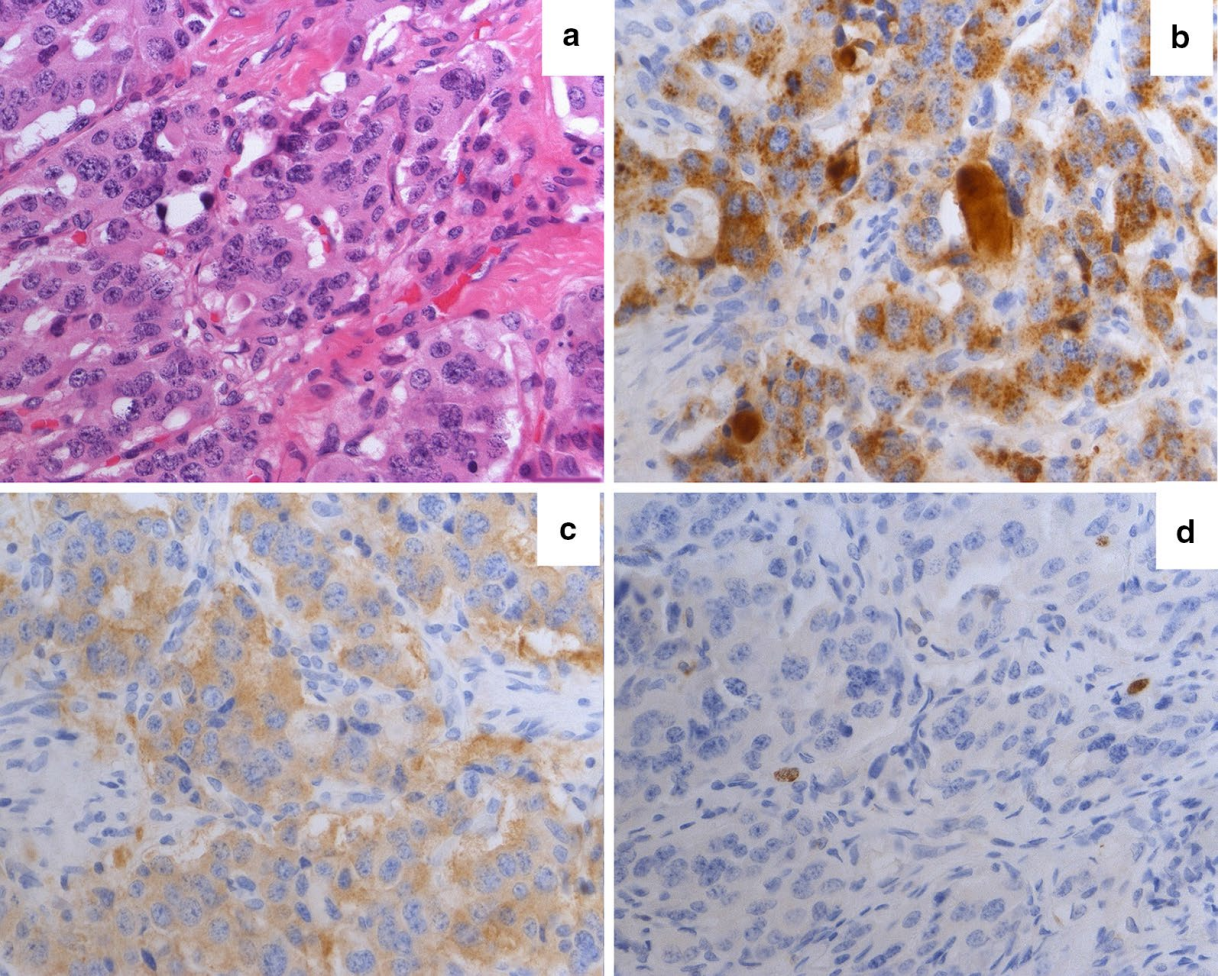
Neuroendocrine tumor with typical growth pattern. Tumor cells show intermediate cytological pleomorphy and a distinct ‘‘pepper and salt’’ chromatin of the nucleolus as well as details of the so called “Zellballen” or nesting pattern (**a**). Antibodies directed against chromogranin **a** show a strong staining pattern (**b**) while synaptophysin is intermediately expressed (**c**). Immunohiostochemical staining with antibodies against Ki-67 indicate a low proliferation rate <2 % (**d**)

**Fig. 4:**
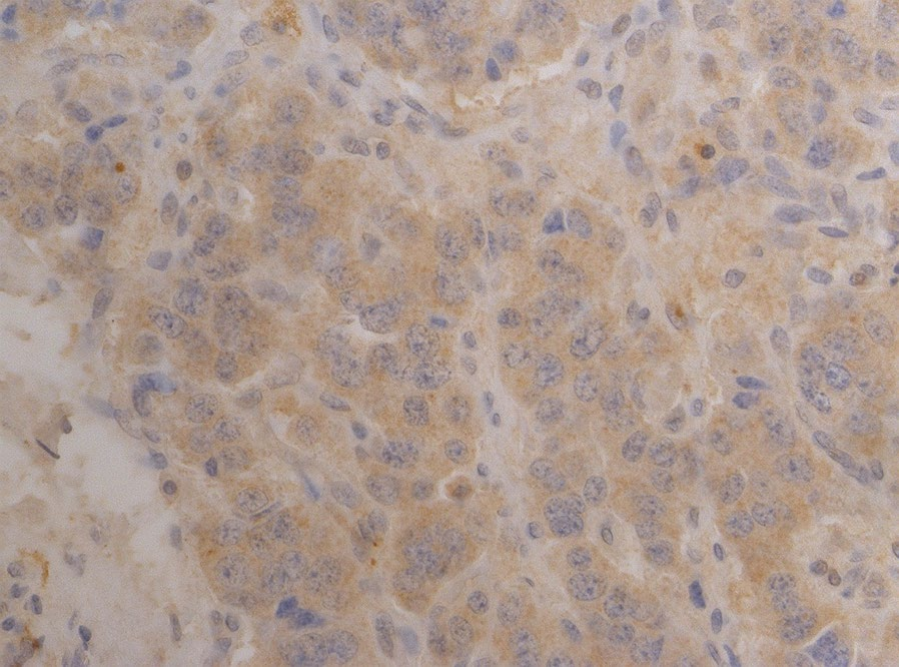
Abundant cytoplasmic protein expression of IGF-1 in the resected tumor specimen as detected by immunohistochemistry

Three years before presenting at our ENETS center, when he was 43 years old, the patient was diagnosed with a WHO grade 1 subependymoma. This intracranial lesion was resected microsurgically in an external hospital. Further pre-existing conditions were unknown. After surgery the patient complained about dyspnea and cough. An X-ray examination at that time revealed a right-sided basal pneumonia, which was treated with antibiotics. However, during the following years, the patient suffered from an abnormal feeling of satiety, weight gain of 20 kg in 3 years and acral enlargement (Fig. 1). His shoe size increased from 44 to 46, the size of gloves from 9 to 11. Furthermore, he developed pain in his ankles, knees as well as in his hips and femur. He noticed a protrusion of the jaw and a clipped speech due to macroglossia and sleeping apnea. Repeated contrast-enhanced imaging studies were performed to rule out pathologies at the level of the pituitary gland. These examinations did not reveal any evidence of a pituitary adenoma. However, growth hormone (GH) and insulin-like growth factor 1 (IGF-1) levels were found to be massively elevated (Table 1). In contrast, growth hormone-releasing hormone (GHRH) was not elevated. GH suppression testing showed clearly the presence of a clinically active acromegaly. A subsequent ^68^Gallium-DOTATOC PET-CT revealed a large tumor-atelectasis-complex in the right basal upper lobe with a compression of the middle and lower bronchus with somatostatin receptor positivity indicative for a well-differentiated neuroendocrine pulmonary tumor (Fig. 2). Retrospectively, a tumor formation could be assumed when comparing X-ray images of 2010 with actual PET-CT. Metastatic lesions were not detected by PET-CT indicating a localized disease stage. Subsequently, endobronchial ultrasound with transbronchial needle aspiration was performed. The tissue was analyzed immunohistochemically revealing an intense positive staining for synaptophysin, chromogranin A and CD 56 with a small amount of Ki-67 positive cells (Fig. 3). In contrast, TTF-1 was negative. Following interdisciplinary tumor board recommendation, the patient has been resected in curative intent and lobectomy was performed. Afterwards, immunohistochemical evaluations presented a typical pulmonary carcinoid (Ki-67 < 2 %) with abundant IGF-1 expression as cause of the acromegaly (Figs. 3 and 4). Postoperative blood tests confirmed the normalization of circulating GH and IGF-1 as markers of complete removal of the tumor (Table 1). Clinically, the patient improved dramatically. Analysis of the coding region of the MEN 1-gen (Exon 2–10; Chromosome 11q13) and flanking intron regions did not reveal any aberration of the sequence or deletion/duplication of single exons. Moreover, also the family history of the patient did not present the diagnosis of a neuroendocrine neoplasia. To date, the patient is recurrence-free and under follow-up.

**Table 1:** Pre- and post-surgery biomarkers for acromegaly

	Standard	Pre-surgery 03/2014	Post-surgery 09/2014
GH in ng/ml	<0.8	10.2	0.3
IGF-1 in µm/l	101–267	1294	261
CgA in U/l	<18	164	31
HbA1c in %	<6	6.8	6.0

## Discussion

### Pulmonary neuroendocrine tumors

Up to 2 % of all lung cancers are pulmonary neuroendocrine tumors [2, 4, 10]. 25 % of all neuroendocrine tumors are located in the respiratory tract what makes them the most frequent extraintestinal site for NET [11]. The reported annual incidence of pulmonary NET equals about 1.35/100,000 [12]. These malignancies form a heterogeneous subset of neoplasias including typical (TC) and atypical carcinoid (AC) tumors, large cell neuroendocrine carcinoma (LCLC) and the most frequently occurring small cell lung cancer (SCLC) which is mostly considered as separate entity. Poorly differentiated neuroendocrine carcinomas display a highly distinct phenotype compared to pulmonary NET in terms of prevalence, biology and prognosis. The following discussion will focus on the typical and atypical carcinoid tumors, since poorly differentiated neuroendocrine carcinomas are characterized by a grossly different biological behavior and are associated with a poor prognosis. Pulmonary NET frequently develop in the bronchial system. Therefore, they likely cause coughing, wheezing, hemoptysis and recurrent pneumonia [13]. If metastasized, typical have a considerably better prognosis compared to atypical NET (15 vs. 50 % risk of metastatic disease by first presentation). According to prior published series, 5- and 10-year survival rates are 80 % for TC (both time points) and 50 and 35 % for AC stage [1, 14].

### Paraneoplastic endocrine syndromes (ectopic hormone production)

Paraneoplastic endocrine syndromes (PES) are caused by tumors capable of producing a variety of functionally active peptides that imitate hormone function and lead to a metabolic disturbance [15]. Tumors derived from tissues that normally do not produce hormones lead to “ectopic” or “inappropriate” hormone production [16]. Neoplastic dedifferentiation can possibly activate repressed genes. That is the reason why this phenomenon most frequently arises in dedifferentiated tumors such as small cell lung cancer [17]. Commonly occurring PES are ACTH or CRH production leading to cushing syndrome, hypercalcemia caused by parathyroid hormone-related protein or syndrome of inappropriate secretion of antidiuretic hormone (SIADH).

In our case the patient presented with the rare paraneoplastic manifestation of acromegaly. Acromegaly represents a rare condition usually affecting middle-aged adults with a prevalence of 40–70 cases per million and an incidence rate of 3–4 cases per million per year [18–21]. This disease mostly results from persistent hypersecretion of growth hormone (GH) leading to hepatic overproduction of insulin-like growth factor-1 (IGF-1). However, both hypersecretion of growth hormone-releasing hormone (GHRH) and growth hormone (GH) may lead to acromegaly. These hormones are produced by the pituitary gland or non-pituitary tumors rarely occur in association with familial syndromes. The vast majority of about 90 % of the patients with acromegaly have a benign pituitary adenoma with monoclonal GH secretion whereof 70 % are macroadenomas (>1 cm) at diagnosis [22, 23]. Ectopic secretion of GH has only been described in single cases [24, 25]. Non-pituitary tumors such as small-cell lung cancer, adrenal adenoma, medullary thyroid carcinoma or pheochromocytoma or pancreatic neuroendocrine tumors can lead to acromegaly by production of growth hormone–releasing hormone (GHRH) [26]. Peripheral production of GHRH causes hyperstimulation of somatotroph cells with subsequently increased GH secretion and hepatic IGF-1 release. Ectopic acromegaly represents less than 1 % of the reported cases of acromegaly [27]. The most common signs are—as the name suggests—acral and soft tissue overgrowth and the typical coarse facies. Acral enlargement is typically noticed by tightness of rings or an increasing shoe size. As physical changes occur gradually clinical diagnosis is often delayed. If acromegaly is suspected IGF-1 measurement and a GH suppression test will be performed. Imaging helps to pinpoint the location and the size of a suspected tumor. Cardiac and respiratory disorders along with arthropathy rank amongst the most frequent systemic complication that lead to increased morbidity and mortality [28, 29]. Patients benefit extensively from controlling GH and IGF-1 hypersecretion either surgically or by pharmacotherapy [30]. This can effectively be achieved by somatostatin. Long acting somatostatin analogs may control ectopic hormonal secretion syndrome, and restrict tumor growth [31].

Single cases of neuroendocrine tumors causing acromegaly have been described with tumors deriving from duodenum [32], liver [33] and from the pancreas in the context of multiple endocrine neoplasia type 1 [9, 34, 35]. In addition, acromegaly due to GHRH hypersecretion in carcinoid tumors is the most common cause [27, 36, 37], whereas paraneoplastic GH secretion is extremely rare in NET [24].

In summary, the case presented here is an extremely rare example of a sporadic pulmonary NET associated with GH and IGF-1 but not GHRH secretion, thereby inducing a clinically florid acromegaly. Thus, mostly inapparent pulmonary NET induce relevant symptoms which should accurately be clarified.

## Methods

We retrospectively analyzed a patient with a pulmonary NET treated and followed-up in our institution since 2013. This case presentation was conducted in accordance with the Declaration of Helsinki. Tumor tissue was explored immunohistochemically concerning expression of chromogranin A, synaptophysin and Ki-67. Analyses were performed according to a standardized protocol using Leica-Bond-Max-Autostainer and the antibodies in the following dilutions: Chromogranin: Dako 1:400; Synaptophysin: Dako 1:400; Ki-67: Dako 1:1000. For IGF-1 we used the ABCM ab 40,657 in the following dilution: 1:400.

The CARE guidelines were followed in accordance with the journal policies.

## Abbreviations

NET: neuroendocrine tumor
NEN: neuroendocrine neoplasia
GH: growth hormone
GHRH: growth hormone-releasing hormone
IGF-1: insulin-like growth factor 1
TC: typical carcinoid
AT: atypical carcinoid
LCLC: large cell neuroendocrine carcinoma
SCLC: small cell lung cancer
PES: paraneoplastic endocrine syndromes
CgA: chromogranin A

## References

[1] Yao JC, Hassan M, Phan A, Dagohoy C, Leary C, Mares JE, Abdalla EK, Fleming JB, Vauthey JN, Rashid A (2008). One hundred years after carcinoid: epidemiology of and prognostic factors for neuroendocrine tumors in 35,825 cases in the United States. J Clin Oncol.

[2] Naalsund A, Rostad H, Strøm EH, Lund MB, Strand TE (2011). Carcinoid lung tumors–incidence, treatment and outcomes: a population-based study. Eur J Cardiothorac Surg.

[3] Hörsch D, Schmid KW, Anlauf M, Darwiche K, Denecke T, Baum RP, Spitzweg C, Grohé C, Presselt N, Stremmel C (2014). Neuroendocrine tumors of the bronchopulmonary system (typical and atypical carcinoid tumors): current strategies in diagnosis and treatment. Conclusions of an expert meeting February 2011 in Weimar, Germany. Oncol Res Treat.

[4] Caplin ME, Baudin E, Ferolla P, Filosso P, Garcia-Yuste M, Lim E, Oberg K, Pelosi G, Perren A, Rossi RE (2015). Pulmonary neuroendocrine (carcinoid) tumors: european Neuroendocrine Tumor Society expert consensus and recommendations for best practice for typical and atypical pulmonary carcinoids. Ann Oncol.

[5] Oliveira AM, Tazelaar HD, Wentzlaff KA, Kosugi NS, Hai N, Benson A, Miller DL, Yang P (2001). Familial pulmonary carcinoid tumors. Cancer.

[6] Lou F, Sarkaria I, Pietanza C, Travis W, Roh MS, Sica G, Healy D, Rusch V, Huang J (2013). Recurrence of pulmonary carcinoid tumors after resection: implications for postoperative surveillance. Ann Thorac Surg.

[7] Travis WD, Gal AA, Colby TV, Klimstra DS, Falk R, Koss MN (1998). Reproducibility of neuroendocrine lung tumor classification. Hum Pathol.

[8] Kaltsas G, Androulakis II, de Herder WW, Grossman AB (2010). Paraneoplastic syndromes secondary to neuroendocrine tumours. Endocr Relat Cancer.

[9] Biermasz NR, Smit JW, Pereira AM, Frölich M, Romijn JA, Roelfsema F (2007). Acromegaly caused by growth hormone-releasing hormone-producing tumors: long-term observational studies in three patients. Pituitary.

[10] Chen LC, Travis WD, Krug LM (2006). Pulmonary neuroendocrine tumors: what (little) do we know?. J Natl Compr Canc Netw.

[11] Öberg K, Hellman P, Ferolla P, Papotti M, Group EGW (2012). Neuroendocrine bronchial and thymic tumors: ESMO Clinical Practice Guidelines for diagnosis, treatment and follow-up. Ann Oncol.

[12] Modlin IM, Lye KD, Kidd M (2003). A 5-decade analysis of 13,715 carcinoid tumors. Cancer.

[13] Kulke MH (2007). Clinical presentation and management of carcinoid tumors. Hematol Oncol Clin North Am.

[14] Travis WD, Rush W, Flieder DB, Falk R, Fleming MV, Gal AA, Koss MN (1998). Survival analysis of 200 pulmonary neuroendocrine tumors with clarification of criteria for atypical carcinoid and its separation from typical carcinoid. Am J Surg Pathol.

[15] DeLellis RA, Xia L (2003). Paraneoplastic endocrine syndromes: a review. Endocr Pathol.

[16] Baylin SB, Mendelsohn G (1980). Ectopic (inappropriate) hormone production by tumors: mechanisms involved and the biological and clinical implications. Endocr Rev.

[17] Gandhi L, Johnson BE (2006). Paraneoplastic syndromes associated with small cell lung cancer. J Natl Compr Canc Netw.

[18] Holdaway IM, Rajasoorya C (1999). Epidemiology of acromegaly. Pituitary.

[19] Bengtsson BA, Edén S, Ernest I, Odén A, Sjögren B (1988). Epidemiology and long-term survival in acromegaly. A study of 166 cases diagnosed between 1955 and 1984. Acta Med Scand.

[20] Scacchi M, Cavagnini F (2006). Acromegaly. Pituitary.

[21] Ribeiro-Oliveira A, Barkan A (2012). The changing face of acromegaly—advances in diagnosis and treatment. Nat Rev Endocrinol.

[22] Sanno N, Teramoto A, Osamura RY, Horvath E, Kovacs K, Lloyd RV, Scheithauer BW (2003). Pathology of pituitary tumors. Neurosurg Clin N Am.

[23] Scheithauer BW, Kurtkaya-Yapicier O, Kovacs KT, Young WF, Lloyd RV (2005). Pituitary carcinoma: a clinicopathological review. Neurosurgery.

[24] Melmed S, Ezrin C, Kovacs K, Goodman RS, Frohman LA (1985). Acromegaly due to secretion of growth hormone by an ectopic pancreatic islet-cell tumor. N Engl J Med.

[25] Beuschlein F, Strasburger CJ, Siegerstetter V, Moradpour D, Lichter P, Bidlingmaier M, Blum HE, Reincke M (2000). Acromegaly caused by secretion of growth hormone by a non-Hodgkin’s lymphoma. N Engl J Med.

[26] Thorner MO, Perryman RL, Cronin MJ, Rogol AD, Draznin M, Johanson A, Vale W, Horvath E, Kovacs K (1982). Somatotroph hyperplasia. Successful treatment of acromegaly by removal of a pancreatic islet tumor secreting a growth hormone-releasing factor. J Clin Invest.

[27] Fainstein Day P, Frohman L, Garcia Rivello H, Reubi JC, Sevlever G, Glerean M, Fernandez Gianotti T, Pietrani M, Rabadan A, Racioppi S (2007). Ectopic growth hormone-releasing hormone secretion by a metastatic bronchial carcinoid tumor: a case with a non hypophysial intracranial tumor that shrank during long acting octreotide treatment. Pituitary.

[28] Colao A, Pivonello R, Marzullo P, Auriemma RS, De Martino MC, Ferone D, Lombardi G (2005). Severe systemic complications of acromegaly. J Endocrinol Invest.

[29] Isgaard J, Arcopinto M, Karason K, Cittadini A (2015). GH and the cardiovascular system: an update on a topic at heart. Endocrine.

[30] Arosio M, Reimondo G, Malchiodi E, Berchialla P, Borraccino A, De Marinis L, Pivonello R, Grottoli S, Losa M, Cannavò S (2012). Predictors of morbidity and mortality in acromegaly: an Italian survey. Eur J Endocrinol.

[31] Doga M, Bonadonna S, Burattin A, Giustina A (2001). Ectopic secretion of growth hormone-releasing hormone (GHRH) in neuroendocrine tumors: relevant clinical aspects. Ann Oncol.

[32] Colak Ozbey N, Kapran Y, Bozbora A, Erbil Y, Tascioglu C, Asa SL (2009). Ectopic growth hormone-releasing hormone secretion by a neuroendocrine tumor causing acromegaly: long-term follow-up results. Endocr Pathol.

[33] Furrer J, Hättenschwiler A, Komminoth P, Pfammatter T, Wiesli P (2001). Carcinoid syndrome, acromegaly, and hypoglycemia due to an insulin-secreting neuroendocrine tumor of the liver. J Clin Endocrinol Metab.

[34] Weiss DE, Vogel H, Lopes MB, Chang SD, Katznelson L (2011). Ectopic acromegaly due to a pancreatic neuroendocrine tumor producing growth hormone-releasing hormone. Endocr Pract.

[35] Sala E, Ferrante E, Verrua E, Malchiodi E, Mantovani G, Filopanti M, Ferrero S, Pietrabissa A, Vanoli A, La Rosa S (2013). Growth hormone-releasing hormone-producing pancreatic neuroendocrine tumor in a multiple endocrine neoplasia type 1 family with an uncommon phenotype. Eur J Gastroenterol Hepatol.

[36] Butler PW, Cochran CS, Merino MJ, Nguyen DM, Schrump DS, Gorden P (2012). Ectopic growth hormone-releasing hormone secretion by a bronchial carcinoid tumor: clinical experience following tumor resection and long-acting octreotide therapy. Pituitary.

[37] Lock KY, Lau IT, Yeung CK, Chan CP (2014). An unusual cause of acromegaly. Hong Kong Med J.

